# The Application of I-Scan Imaging for Evaluating Benign Vocal Lesions

**DOI:** 10.3390/diagnostics14030270

**Published:** 2024-01-26

**Authors:** Che-Hsien Chou, Chih-Hua Chen, Andy Wei-Ge Chen

**Affiliations:** 1 Department of Speech Language Pathology and Audiology, Chung Shan Medical University, Taichung 402, Taiwan; s0880069@gm.csmu.edu.tw; 2 Department of Otorhinolaryngology, Head and Neck Surgery, Changhua Christian Hospital, Changhua 500, Taiwan; 79007@cch.org.tw; 3 Doctoral Program in Translational Medicine, National Chung Hsing University, Taichung 402, Taiwan; 4 Rong Hsing Translational Medicine Research Center, National Chung Hsing University, Taichung 402, Taiwan

**Keywords:** i-scan, vocal polyp, vocal nodule, vocal cyst

## Abstract

Current standard methods for evaluating benign vocal lesions, including white light laryngoscopy and video laryngostroboscopy, may struggle to identify smaller lesions. While histopathological results obtained from laryngeal microsurgery provide definitive results, their invasiveness can lead to scarring and impaired phonological outcomes. Intralesional steroid injection has recently gained acceptance, but it lacks pathological diagnostic capabilities. Therefore, there is a growing need for a simple examination that can enhance the diagnosis of benign vocal lesions. NBI, from Olympus Corporation, has shown promising outcomes in detecting and characterizing laryngeal lesions. The i-scan technology by PENTAX, while providing the ability to improve visual clarity during endoscopic procedures, has been addressed less in this field. Our study aims to further investigate the application of i-scan imaging in benign vocal lesions, enrolling patients diagnosed with vocal cysts, polyps, and nodules. We conducted i-scan imaging prior to office-based intralesional steroid injection, assessing the possibility of its providing additional diagnostic information for benign vocal lesions without additional burden.

**Figure 1:**
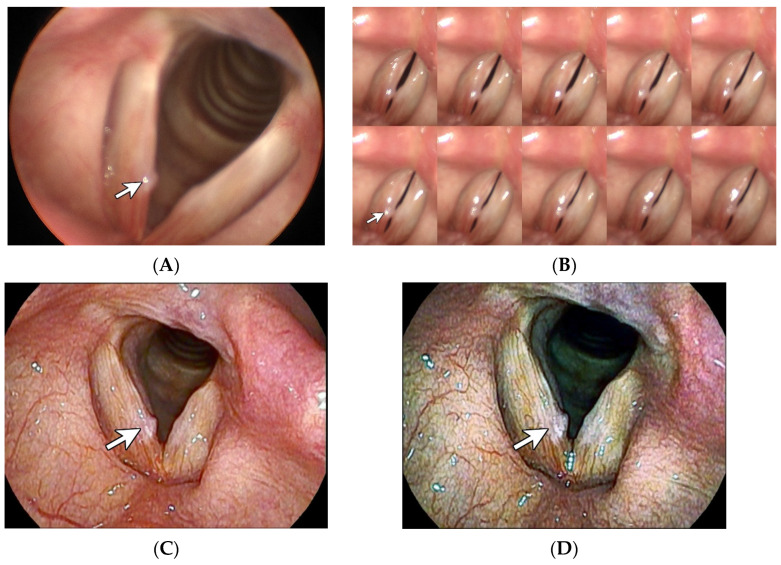
Vocal nodule. A 59-year-old female factory worker had been experiencing hoarseness for over 1 year. Stills taken from laryngostroboscopy (**A**) revealed a tiny whitish sessile lesion, which is labeled with a white arrow, accompanied by a reactive lesion on the contralateral vocal fold. The montage 10-frame analysis from the laryngostroboscopy (**B**) revealed symmetrical glottal cycles that were characterized by incomplete closure, featuring an hourglass-shaped glottal configuration, which might be comparable to vocal nodules. White light laryngoscopy with CE + 2 (**C**) revealed the presence of an irregular edge of the lesion. I-scan imaging [1] with SE + 5, CE + 2, and TE-c modes (**D**) further highlighted the irregular edge. Meanwhile, the characteristics of stiff surface, thicker epithelium, and less vascularization were clearly enhanced into a rough whitish appearance. Taken together, these findings serve to strengthen the diagnosis of vocal nodules in this patient.

**Figure 2:**
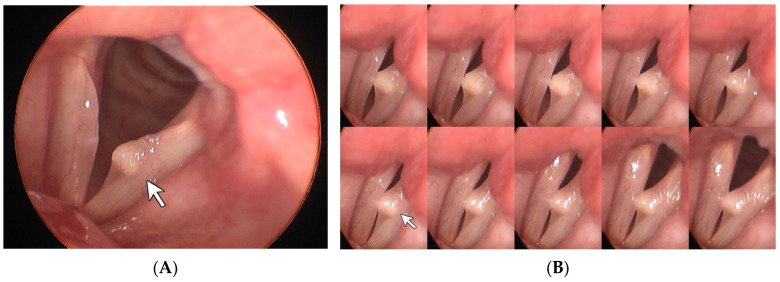

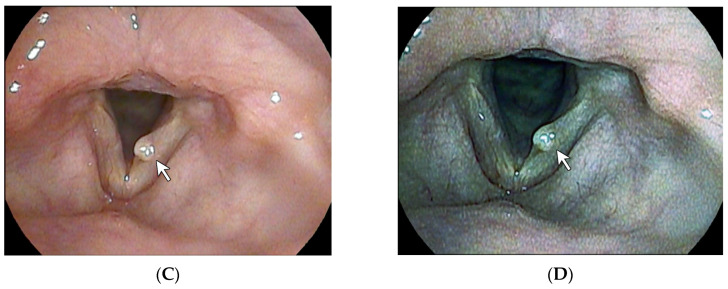
Vocal cyst. A 41-year-old female full-time homemaker had been experiencing hoarseness for over 1 year. Stills taken from laryngostroboscopy (**A**) revealed a round lesion, which is labeled with a white arrow. The montage 10-frame analysis from the laryngostroboscopy (**B**) further showed an absence of mucosal wave and incomplete closure. White light laryngoscopy with CE + 2 (**C**) highlighted the smooth and round edge of the lesion. Under i-scan imaging (**D**), the epithelial lining of the lesion was enhanced into a whitish appearance and the margin of the sac-like structure was clearly outlined. Based on these findings, a vocal cyst can be reasonably diagnosed in this case.

**Figure 3:**
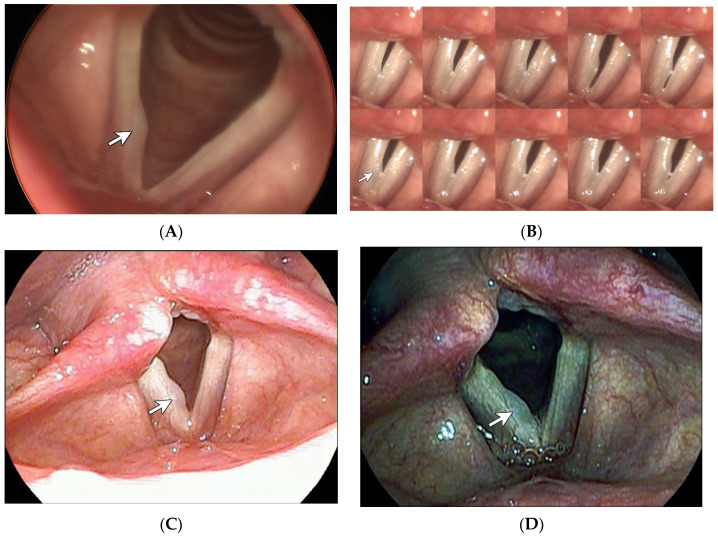
Vocal polyp. A 43-year-old female teacher had been experiencing hoarseness for over one year. Stills taken from the laryngostroboscopy (**A**) revealed a thin broad lesion labeled with a white arrow. The montage 10-frame analysis from the laryngostroboscopy (**B**) demonstrated a generally intact mucosal wave.White light laryngoscopy with CE + 2 (**C**) highlighted the broad, smooth, and convex edge of the lesion. Under i-scan imaging (**D**), the lesion was demonstrated in a darker and translucent fashion. This is comparable to the fact that vocal polyps generally exhibit a thinner epithelium and more prominent vascularization when compared to vocal nodules [2]. Taken together, vocal polyps can be reasonably diagnosed in this case.
